# An evaluation of the usefulness of single pass albumin dialysis: key role of dialysate flow rate

**DOI:** 10.1186/s13054-016-1287-4

**Published:** 2016-05-26

**Authors:** Mariusz Piechota, Anna Piechota

**Affiliations:** Department of Anaesthesiology and Intensive Therapy, Centre for Artificial Extracorporeal Kidney and Liver Support, Dr Wł. Biegański Regional Specialist Hospital, Kniaziewicza 1/5, Łódź, 91-347 Poland; Department of Insurance, Faculty of Economics and Sociology, University of Łódź, Łódź, Poland

In their article in *Critical Care*, Sponholz et al. [1] compared two devices: the molecular adsorbent recirculating system (MARS) and single pass albumin dialysis (SPAD). As Sponholz et al. claim, SPAD is not an effective method for achieving a significant decrease in the concentration of bile acids, creatinine or urea, because a single SPAD procedure induces a 7.62 % (median) reduction in the level of bile acids and an increase in the concentrations of creatinine and urea by respectively 5.04 % (median) and 4.69 % (median), compared with pre-dialysis levels [1]. In contrast, our as yet unpublished study demonstrates that SPAD can effectively reduce not only the level of bile acids but also the concentration of ammonia. Based on our study, the application of the SPAD method with a dialysate flow rate of 1000 ml/h for a total of around 10 h reduces the level of bile acids by 21.9 % (median) and ammonia by 16.25 % (median).

A question arises as to the possible causes of such discrepancies. Since continuous venovenous haemodialysis was performed in both studies, the divergence cannot be attributed to the technique. In our view, this divergence is associated with several factors. One of these factors is the difference in dialysate flow rate. An albumin dialysate flow rate of 700 ml/h is highly insufficient. Taking into consideration the results obtained by Sponholz et al., an increase in the albumin dialysate flow rate to at least 1000 ml/h appears to be a necessary precondition to ensure effective elimination of bile acids. Also, the methodology adopted for the study is questionable. In our opinion, comparing the effectiveness of urea and creatinine elimination at radically different dialysate flow rates (SPAD 700 ml/h vs MARS 2000 ml/h) is unjustified and may lead to erroneous conclusions, which in fact occurred [1].

Differences in efficacy between the MARS and SPAD procedures reported in the article in question may also be related to inappropriately selected haemofilters in the extracorporeal blood purification systems under comparison.

Furthermore, Sponholz et al. [1] pointed out metabolic derangements and electrolyte disturbances, particularly in SPAD using regional citrate anticoagulation. In our view, increasing the dialysate flow rate would significantly contribute to preventing the irregularities noted in the study. On the other hand, we believe that the risk of citrate overdose during liver dialysis, but also during continuous venovenous haemodialysis, is very low [2–4]. In our medical centre, we have been successfully performing continuous venovenous haemodialysis procedures with regional citrate anticoagulation in patients with severe liver dysfunction, sometimes for up to several weeks. We have never noted any symptoms of overdose apart from end-stage liver failure.

## Abbreviations

MARS: molecular adsorbent recirculating system
SPAD: single pass albumin dialysis

## References

[1] Sponholz C, Matthes K, Rupp D, Backaus W, Klammt S, Karailieva D (2016). Molecular adsorbent recirculating system and single-pass albumin dialysis in liver failure—a prospective, randomised crossover study. Crit Care..

[2] Slowinski T, Morgera S, Joannidis M, Henneberg T, Stocker R, Helset E (2015). Safety and efficacy of regional citrate anticoagulation in continuous venovenous hemodialysis in the presence of liver failure: the Liver Citrate Anticoagulation Threshold (L-CAT) observational study. Crit Care..

[3] Faybik P, Hetz H, Mitterer G, Krenn CG, Schiefer J, Funk GC, et al. Crit Care Med. 2011;39:273–9. 10.1097/CCM.0b013e3181fee8a4 20975551

[4] Piechota M (2014). Hepatic encephalopathy in the course of alcoholic liver disease—treatment options in the intensive care unit. Anaesthesiol Intensive Ther..

[5] Sauer IM, Goetz M, Steffen I, Walter G, Kehr DC, Schwartlander R (2004). In vitro comparison of the molecular adsorbent recirculation system (MARS) and single-pass albumin dialysis (SPAD). Hepatol Baltim Md..

[6] Kortgen A, Rauchfuss F, Götz M, Settmacher U, Bauer M (2009). Albumin dialysis in liver failure: comparison of molecular adsorbent recirculating system and single pass albumin dialysis—a retrospective analysis. Ther Apher Dial..

[7] Sponholz C, Settmacher U, Bauer M, Kortgen A (2015). Regional citrate anticoagulation for continuous renal replacement therapy in the perioperative care of liver transplant recipients: a single center experience. Ther Apher Dial..

[8] Sponholz C, Bayer O, Kabisch B, Wurm K, Ebert K, Bauer M (2014). Anticoagulation strategies in venovenous hemodialysis in critically ill patients: a five-year evaluation in a surgical intensive care unit. Sci World J..

