# Supplementary material for: An evaluation of the usefulness of single pass albumin dialysis: key role of dialysate flow rate
Source: Crit Care. 2016 May 26;20:156. doi: 10.1186/s13054-016-1287-4 (PMC4937575; doi:10.1186/s13054-016-1287-4)
Supplement: Related texts

# Reply: Authors’ response

**Authors:** Christoph Sponholz, Steffen Mitzner, Michael Bauer, Andreas Kortgen

**Affiliations:** Department of Anaesthesiology and Critical Care Medicine, Jena University Hospital, Jena, Germany; Division of Nephrology, Department of Medicine, Rostock University Medical Centre, Rostock, Germany; Fraunhofer Institute for Cell Therapy and Immunology, Extracorporeal Immunomodulation Project Group, Rostock, Germany; Center for Sepsis Control and Care, Integrated Treatment and Research Center, Jena University Hospital, Jena, Germany

Corresponding author

Andreas Kortgen

Department of Anaesthesiology and Critical Care Medicine, Jena University Hospital, Erlanger Allee 101

07747 Jena, Germany.

Phone: +49 3641 9 – 32 31 84

eMail: Andreas.kortgen@med.uni-jena.de

We appreciate the interest of Piechota and Piechota in our study. Indeed, SPAD is not a standardised method either with respect to the albumin concentration in the dialysate solution or to dialysate flow rates. The rationale for using dialysis flow rates of 700 ml/h in our study was based on previously published data on the effective detoxification capacity of a 4 % albumin-containing dialysis solution in vitro [5] and our own experience in critically ill patients [6]. Moreover, the flow rate of 700 ml/h allows 7 h of treatment duration similar to MARS using one 5000 ml dialysis bag, resulting in significant changes in total bilirubin levels. However, neither bile acids nor creatinine or urea levels could be reduced in this setting. As discussed extensively, enhancing the dialysis flow rates and treatment times in SPAD could potentially lead to higher detoxification capacities accompanied by higher treatment costs, particularly for higher amounts of albumin. With respect to treatment times the same holds true for MARS, when using a new treatment kit.

Furthermore, Piechota and Piechota raised the question of metabolic and electrolyte disturbances during SPAD administration and citrate anticoagulation in our study. Indeed, we believe that these disturbances were related to the dialysis flow rate, resulting in relative citrate overdosing, and discussed this issue extensively in our publication [1]. Increasing the dialysis flow rate could reduce the risk of citrate overdosing and accumulation. We demonstrated that regional citrate administration can be used effectively and safely in the critical care setting even in patients with severe liver dysfunction [7, 8].

We agree with Piechota and Piechota that the dialysis haemofilter impacts on efficacy of SPAD. Reviewing the literature, successful SPAD procedures were published running with different haemofilters, including polysulfone filters as used in the current study (AV1000s polysulfone ultraflux haemofilter; Fresenius Medical Care, Bad Homburg, Germany). Thus, the haemofilter is unlikely responsible for our findings.

To summarise, the SPAD procedure is published to run at different albumin concentrations, dialysis modes and flow rates and with different haemofilters. Currently little is known about the efficacy of SPAD running in different modalities and no review is available summarising all of these issues. Our study thus represents one piece in the puzzle for the discussion of evaluating and standardising the SPAD procedure.

Moreover, our study underlines that the often-performed clinical routine of only assessing the course of bilirubin levels to evaluate efficacy of extracorporeal liver support is insufficient.
